# Duodenal plasmablastic lymphoma in an human immunodeficency virus-negative patient: a case report

**DOI:** 10.1186/s13256-023-04143-1

**Published:** 2023-10-01

**Authors:** Abbas Ali Hussain, Aresha Masood Shah, Sumeet Kumar

**Affiliations:** 1 Jinnah Postgraduate Medical Center JPMC, Karachi, Pakistan; 2 Dow Medical College, Karachi, Pakistan

**Keywords:** Plasmablastic lymphoma, Duodenal tumour, Non-Hodgkin lymphoma, HIV, PBL

## Abstract

**Background:**

Plasmablastic lymphoma is a rare type of non-Hodgkin lymphoma that generally presents an aggressive clinical course. It is strongly associated with human immunodeficency virus (HIV) infection, and the most common site of involvement is the oral cavity. Although extraoral PBL has been reported in several places, small intestine involvement is extremely rare.

**Case presentation:**

Here, we describe an exceptionally rare case of a 24-year-old immunocompetent Asian Male patient with newly diagnosed plasmablastic lymphoma of the duodenum. The patient was admitted to our oncology facility due to the patient's clinical course, which included persistent vomiting, hematemesis, weight loss, and generalized weakness. Computed tomography of the abdomen (triphasic) of the patient showed thickness at the 2nd part of the duodenum measuring 2.6 cm in width and 16 cm in length blocking the pancreatic and common bile ducts by entering the second section of the duodenum. The biopsy specimen's pathological investigation indicated abnormal cells with plasmacytoid characteristics and a high proliferation index. The diagnosis of PBL was confirmed by immunohistochemical profiling. Supportive therapies like blood transfusions, antacids, and antiemetics were started to manage the patient's symptoms. Palliative radiation was also anticipated for the lesion site.

**Conclusions:**

Duodenal involvement to the extent seen in our patient is exceptionally rare and, to the best of our knowledge, has hardly been described. The main goal of the article is to review the literature and report a case.

## Introduction

Plasmablastic lymphoma (PBL) is a rare variant of diffuse large B-cell lymphoma (DLBCL) that is aggressive in nature and pathologically similar to plasma cell myeloma [1]. PBL was initially described in the oral cavity in patients with human immunodeficency virus (HIV) infection, but with improved awareness of the disease and a better understanding of its biology, PBL was subsequently identified in HIV negative individuals [2]. Extraoral PBL involving the lungs, nasal cavity, lymph nodes and skin has been reported in various case reports; however, PBL of the small intestine is extremely rare [3]. Here, we present a rare case of duodenal plasmablastic lymphoma in a 24-year-old HIV-negative patient.

## Case presentation

A 24-year-old Asian Male patient was admitted to the clinical oncology ward of Jinnah Postgraduate Medical Center—JPMC, (Karachi, Pakistan) on 23rd August 2021. The patient had a history of intractable vomiting, hematemesis, generalized weakness, weight loss, epigastric pain and melena that had lasted more than a month.

Upon physical examination, the patient seemed underweight and anaemic, a palpable mass in the epigastric region was noted with hepatosplenomegaly felt during abdominal palpation and profound jaundice.

Notably, the patient's medical history did not reveal any recognized risk factors or underlying health issues that would have contributed to the plasmablastic lymphoma's duodenal presentation. There was no prior history of HIV infection or any other impaired conditions.

The initial laboratory workup (Table 1) revealed severe anaemia with low haemoglobin and haematocrit and elevated bilirubin, GGT and ALP. The differential white blood cell and platelet counts, and renal function tests were normal. Serology results for HIV, hepatitis B surface antigen, hepatitis C and H. Pylori IgG were negative.

**Table 1:** Laboratory test results for the patient

Laboratory test	Baseline level	Lab workup	Normal range
Haemoglobin	6.0 g/dl	9.8 g/dl	14–17.5 g/dl
Haematocrit	18.5%	30.1%	42–52%
Total Bilirubin	9.35 mg/dl	12.21 mg/dl	0.3–1.9 mg/dl
Gamma-glutamyl transferase (GGT)	764 IU/L	852 IU/L	0–50 IU/L
Alkaline Phosphatase (ALP)	2310 IU/L	3868 IU/L	44–147 IU/L

Laboratory test results for the patient, showing the haemoglobin, haematocrit, total bilirubin, gamma-glutamyl transferase (GGT), and alkaline phosphatase (ALP) levels. The normal range for each laboratory test is also provided

Esophagogastroduodenoscopy identified irregular circumferential ulcerated growth in the first part of the duodenum (D1) extending up to the junction of the first and second parts of the duodenum (D2). A large clean-based ulcer with an overlying clot was also noted, consistent with Class II-B of the Forrest Classification for peptic ulcers. Biopsy of the ulcerated mass was taken during esophagogastroduodenoscopy for histopathology.

Computed tomography of the abdomen (triphasic) showed thickness at the 2nd part of the duodenum measuring 2.6 cm in width and 16 cm in length. Thickening infiltrating into the medial head of the pancreas resulting in obstruction and dilatation of the pancreatic and common bile duct was also significant. Ill-defined soft tissue density with necrosis at the pelvis and the upper lobe of the right kidney with enlarged para-aortic lymph nodes was noted, which was suggestive of aggressive primary neoplastic lesions.

The pathological analysis was congruent with haematolymphoid neoplasm with plasmacytoid features. Microscopic description of the specimen revealed sheets of atypical cells of medium to large size that exhibit plasmacytoid features with a high proliferation index (Ki-67 raised) and brisk mitotic activity. Tumour cells were positive for LCA, CD79a, Pax-5, Mum-1, and CD138 and negative for CD20, Pan T (CD3) and CD30. The diagnosis of PBL was made by the physicians at JPMC, Karachi. Supportive treatment was started, including 3 pint packed red blood cell (PRBC) blood transfusions to correct anaemia, injection of tranexamic acid 500 mg TDS, omeprazole (40 mg IV OD) and octreotide continuous IV infusion of 25 mcg/hr to control GI bleeding and ondansetron (8 mg IV BD) for vomiting. In addition, palliative radiotherapy (RTP) at the site of the lesion was planned.

The patient was lost to follow-up despite these efforts, which made it difficult to further assess the therapeutic response and long-term results.

## Discussion

Plasmablastic lymphoma remains a diagnostic challenge, as the biological characteristics of the disease are enigmatic to date. The diagnosis of such cancers might be more difficult in the setting of extraoral locations and in immunocompetent patients. As a rare entity, PBL was originally described as a separate clinicopathologic entity by Delecluse *et al.* as a rapidly proliferating B-cell lymphoma occurring in the oral cavity in the context of HIV infection [3]. Although the exact incidence of PBL is not known, it accounts for 2–3% of all AIDS-related lymphomas [1]. Furthermore, the majority of the cases reported arose in HIV-positive individuals, and in recent years, the occurrence of PBL in HIV-negative patients has been reported in escalating numbers [4].

Extraoral sites of PBL, including skin, subcutaneous tissue, stomach, anal mucosa or perianal area, lung and lymph nodes, have been reported in several cases; however, involvement of the small intestines is rare, with only a single case reported of duodenal involvement in the last ten years (January 2010–January 2020) [5, 6]. The HIV status of the reported case was negative, similar to the disease occurrence we describe in our study. These findings establish the uniqueness of this study and hence an area to be researched thoroughly for future disease diagnosis and management.

In 2014, a case series reported the manifestation of gastrointestinal plasmablastic lymphoma (GI-PBL) in four patients who presented to the Moffit Cancer Center for management. The age at presentation for all four cases was between 40 and 65 years with underlying immunosuppressive statuses such as HIV and EBV positivity [7]. This is not consistent with our data, where the patient was a known case of GI-PBL in the third decade of life with no underlying chronic diseases (HIV and Hepatitis B), thus making him immunocompetent. In addition, Epstein‒Barr virus (EBV) expression was inconclusive, and there was no history of steroid intake, which might reduce immunity. Previous studies also concluded that HIV-negative individuals have lower rates of oral involvement and EBV expression than HIV-positive individuals [2, 8]. Another literature review collected data from 112 cases of PBL regarding patient age and gender, HIV status, initiation of and response to highly active antiretroviral therapy (HAART), and outcome of management. These 112 PBL cases reported HIV-positive status with a median age at presentation of approximately 38 years and a male predominance of 7:1. Two of the patients discussed in this review were 7 and 11 years old with different primary lesions, such as oral cavity and cutaneous involvement, respectively, which is also not in line with our case [9]. In PBL, age and primary lesion site might have a strong correlation to factors other than immune status; therefore, the gaps need to be filled by conducting more research for a better understanding of the association between age, primary lesion, and immune status.

For oncologists, the diagnosis of PBL in immunocompetent and extraoral site localization is very demanding because the clinical, morphological, phenotypic, and molecular features are ambiguous.

In addition, due to the lack of a distinguishing phenotype, differential diagnosis with the activated B-cell-like (ABC-like) subgroup of DLBCL and plasma cell malignancy (PCM) with plasmablastic morphology is still considered a common dilemma. Although there are wide-ranging differential diagnoses including plasmablastic multiple myeloma, Burkitt lymphoma (BL), Hodgkin lymphoma, primary effusion lymphoma and extramedullary plasmablastic myeloma [10–12].

In practice, PBL can be differentiated from BL and immunoblastic DLBCL based on immunohistochemistry (CD20 and LCA immunoreactivity present in BL and absent in PBL) [13, 14]. Plasmablastic myeloma (PCM) and PBL both share cytomorphologic and immunophenotypic features; therefore, differentiation between the two is very challenging. [15] Therefore, Clinical correlation is considered crucial for the diagnosis of plasmablastic PCM over PBL, where hypercalcemia, Bens Jones protein in the urine, lytic lesions of bones, anaemia Favors plasmablastic PCM, and HIV and EBV association Favors PBL [16]. Plasmacytoma can be differentiated from PBL, where the former lacks MYC gene rearrangement. Extramedullary plasmablastic myeloma is also an important differential diagnosis and requires differentiation from PBL since the two entities share different treatment modalities [10].

Narrowing down the working diagnosis might help in ruling out life-threatening or time-critical conditions, thus increasing the overall survival rate of GI PBL patients. Ruling out other differential diagnoses will also guide oncologists in better medical evaluations and treatment plans.

The range of treatment provided to patients with PBL has been substantial and vast, from medical to radiation, depending upon the extent and severity of the disease [11]. A literature search concluded that in patients with localized disease, where the prognosis is already better, radiotherapy plays a significant role. Therefore, in our case, the team opted for palliative radiotherapy at the site of the lesion for a better quality of life and to minimize crisis at the end of life. Durable remission can also be achieved by combined chemotherapy and radiotherapy [17, 18]. Chemotherapeutic regimens that are very well tolerated include etoposide, vincristine, doxorubicin, cyclophosphamide, vincristine, and prednisone (EPOCH), cyclophosphamide, doxorubicin, vincristine, and prednisone (CHOP), or CHOP-like regimens that include drugs such as hyper fractionated cyclophosphamide, vincristine, doxorubicin, dexamethasone, high-dose methotrexate, cytarabine, oncovin, and bleomycin (CHOP) [19]. Chemotherapeutic agents such as CHOP that are less intensive should be provided first to avoid severe adverse effects, and a combined decision should be made between the doctor and the patient weighing all benefits and risks. Lynette Luria *et al.* also favoured the CHOP regimen in GI-PBL with HIV-negative and CD 20-negative IHC patients, as complete remission was achieved after 6 cycles [7]. Less data is available regarding the surgical approach in PBL cases; therefore, more research is required for future trials that consider PBL management [11]. As remission is achieved by induction chemotherapy [7] and hematopoietic stem cell transplantation is also considered a good option in the management of PBL [10], oncologists should conduct trials with this combination for a better cure. The rarity of the disease makes it difficult to manage; thus, the new therapeutic approaches discussed in the literature review should be considered during the management of PBL in the future. These include bortezomib (protease inhibitor), lenalidomide [20], and adoptive cellular therapies, where tumour-reactive T cells transferred into the cancer patient have a cytotoxic antitumour effect [21]. Controlled studies on PBL management are not currently available; hence, the lack of data in this field further creates many difficulties in increasing the overall survival (OS) of these patients.

## Conclusion

In conclusion, plasmablastic lymphoma is an extremely rare form of HIV-related lymphoma commonly involving the oral cavity; thus, duodenal involvement in an HIV-negative patient makes this possibly the first properly documented case. Plasmablastic lymphoma is an aggressive non-Hodgkin lymphoma; hence, its diagnosis is challenging, particularly when it arises in extraoral locations and in immunocompetent patients. A delayed diagnosis might negatively impact both treatment and survival.

## Data Availability

All data generated or analysed during this study are included in this published article and its supplementary information files.

## References

[1] Nwanwene K, Khan NA, Alsharedi M (2021). Teticular plasmablastic lymphoma in an HIV-negative patient: a rare case presentation. J Investig Med High Impact Case Rep.

[2] Castillo JJ, Winer ES, Stachurski D, Perez K, Jabbour M, Milani C, Colvin GA, Butera JN (2011). HIV-negative plasmablastic lymphoma: not in the mouth. Clin Lymphoma Myeloma Leuk.

[3] Wang HW, Yang W, Sun JZ, Lu JY, Li M, Sun L (2012). Plasmablastic lymphoma of the small intestine: case report and literature review. World J Gastroenterol: WJG.

[4] Liu JJ, Zhang L, Ayala E, Field T, Ochoa-Bayona JL, Perez L, Bello CM, Chervenick PA, Bruno S, Cultrera JL, Baz RC (2011). Human immunodeficiency virus (HIV)-negative plasmablastic lymphoma: a single institutional experience and literature review. Leuk Res.

[5] Koike M, Masuda A, Kunimoto Ichikawa AS, Komatus N (2014). Plasmablastic lasmablastic lymphoma of the duodenal and jejunum. Int J Clin Exp Pathol.

[6] Sanguedolce F, Zanelli M, Zizzo M, Martino G, Rossi C, Parente P, Ascani S (2020). Clinical, pathological and molecular features of plasmablastic lymphoma arising in the gastrointestinal tract: a review and reappraisal. Pathol Res Pract.

[7] Luria L, Nguyen J, Zhou J, Jaglal M, Sokol L, Messina JL, Coppola D, Zhang L (2014). Manifestations of gastrointestinal plasmablastic lymphoma: a case series with literature review. World J Gastroenterol.

[8] Li YJ, Li JW, Chen KL, Li J, Zhong MZ, Liu XL, Yi PY, Zhou H (2020). HIV-negative plasmablastic lymphoma: report of 8 cases and a comprehensive review of 394 published cases. Blood research.

[9] Castillo J, Pantanowitz L, Dezube BJ (2008). HIV-associated plasmablastic lymphoma: lessons learned from 112 published cases. Am J Hematol.

[10] Elyamany G, Al Mussaed E, Alzahrani AM (2015). Plasmablastic lymphoma: a review of current knowledge and future directions. Adv Hematol..

[11] Bhatt R, Desai DS. Plasmablastic Lymphoma. In: StatPearls [Internet]. Treasure Island (FL): StatPearls Publishing; 2023. https://www.ncbi.nlm.nih.gov/books/NBK532975/. Accessed 14 Apr 2022.

[12] Castillo JJ, Bibas M, Miranda RN (2015). The biology and treatment of plasmablastic lymphoma. Blood.

[13] Chetty R, Hlatswayo N, Muc R, Sabaratnam R, Gatter K (2003). Plasmablastic lymphoma in HIV+ patients: an expanding spectrum. Histopathology.

[14] Das DK (2018). Contribution of immunocytochemistry to the diagnosis of usual and unusual lymphoma cases. J Cytol.

[15] Vega F, Chang CC, Medeiros LJ, Udden MM, Cho-Vega JH, Lau CC, Finch CJ, Vilchez RA, McGregor D, Jorgensen JL (2005). Plasmablastic lymphomas and plasmablastic plasma cell myelomas have nearly identical immunophenotypic profiles. Mod Pathol.

[16] Chang CC, Zhou X, Taylor JJ, Huang WT, Ren X, Monzon F, Feng Y, Rao PH, Lu XY, Fabio F, Hilsenbeck S (2009). Genomic profiling of plasmablastic lymphoma using array comparative genomic hybridization (aCGH): revealing significant overlapping genomic lesions with diffuse large B-cell lymphoma. J Hematol Oncol.

[17] Phipps C, Yeoh KW, Lee YS, Nagarajan C, Gopalakrishnan S, Ho LP, Hwang WY, Goh YT, Grigoropoulos NF (2017). Durable remission is achievable with localized treatment and reduction of immunosuppression in limited stage EBV-related plasmablastic lymphoma. Ann Hematol.

[18] Lopez A, Abrisqueta P (2018). Plasmablastic lymphoma: current perspectives. Blood Lymphat Cancer.

[19] Zelenetz AD, Abramson JS, Advani RH, Andreadis CB, Byrd JC, Czuczman MS, Fayad L, Forero A, Glenn MJ, Gockerman JP, Gordon LI (2010). Non-Hodgkin’s lymphomas. J Natl Compr Canc Netw.

[20] Cao C, Liu T, Zhu H, Wang L, Kai S, Xiang B (2014). Bortezomib-contained chemotherapy and thalidomide combined with CHOP (Cyclophosphamide, Doxorubicin, Vincristine, and Prednisone) play promising roles in plasmablastic lymphoma: a case report and literature review. Clin Lymphoma Myeloma Leuk.

[21] Brentjens RJ, Curran KJ (2012). Novel cellular therapies for leukemia: CAR-modified T cells targeted to the CD19 antigen. Hematology Am Soc Hematol Educ Program..

